# Simultaneous *In Vitro* Characterisation of DNA Deaminase Function and Associated DNA Repair Pathways

**DOI:** 10.1371/journal.pone.0082097

**Published:** 2013-12-09

**Authors:** Don-Marc Franchini, Elisabetta Incorvaia, Gopinath Rangam, Heather A. Coker, Svend K. Petersen-Mahrt

**Affiliations:** 1 DNA Editing in Immunity and Epigenetics, IFOM-Fondazione Instituto FIRC di Oncologia Molecolare, Milano, Italy; 2 DNA Editing Lab, Clare Hall Laboratories, London Research Institute, South Mimms, United Kingdom; Université de Montréal, Canada

## Abstract

During immunoglobulin (Ig) diversification, activation-induced deaminase (AID) initiates somatic hypermutation and class switch recombination by catalysing the conversion of cytosine to uracil. The synergy between AID and DNA repair pathways is fundamental for the introduction of mutations, however the molecular and biochemical mechanisms underlying this process are not fully elucidated. We describe a novel method to efficiently decipher the composition and activity of DNA repair pathways that are activated by AID-induced lesions. The in vitro resolution (IVR) assay combines AID based deamination and DNA repair activities from a cellular milieu in a single assay, thus avoiding synthetically created DNA-lesions or genetic-based readouts. Recombinant GAL4-AID fusion protein is targeted to a plasmid containing GAL4 binding sites, allowing for controlled cytosine deamination within a substrate plasmid. Subsequently, the Xenopus laevis egg extract provides a source of DNA repair proteins and functional repair pathways. Our results demonstrated that DNA repair pathways which are in vitro activated by AID-induced lesions are reminiscent of those found during AID-induced in vivo Ig diversification. The comparative ease of manipulation of this in vitro systems provides a new approach to dissect the complex DNA repair pathways acting on defined physiologically lesions, can be adapted to use with other DNA damaging proteins (e.g. APOBECs), and provide a means to develop and characterise pharmacological agents to inhibit these potentially oncogenic processes.

## Introduction

DNA damage encompasses a large variety of chemical and physical alterations of DNA and chromatin. Generated either by endogenous or exogenous sources, DNA lesions can arise from alkylation, oxidation, deamination, depurinations, or are present at single stranded nicks, double strand breaks (DSB), as well as intra- and inter-strand crosslinks [1]. DNA lesions activate DNA damage sensing proteins, which in turn activate and recruit mediators and repair factors, utilising phosphorylation, ubiquitination, sumoylation, and/or poly(ADP)ribosylation as signalling intermediates. This in turn creates an intricate network of protein-protein interactions around the lesion; with each type of lesion inducing the formation of different networks. It has been estimated that every day, every cell experiences 20,000 to 30,000 DNA lesions of various types [2], with DNA repair efficiently processing them and preventing, in most part, genomic instability.

On the other hand, DNA damage is a prerequisite for physiological processes such as immune diversification and meiosis. During B cell development, antigen-dependent immunoglobulin (Ig) diversification requires activation-induced deaminase (AID) to initiate somatic hypermutation (SHM) and class switch recombination (CSR) [3], [4]. AID deaminates cytosines to uracil in single strand DNA (ssDNA), preferring a sequence of WRC (W = A/T, R = A/G) surrounding the cytosine [5]. Upon double strand DNA (dsDNA) formation, the resulting dU:dG mismatch activates proteins belonging to base excision repair (BER) or mismatch repair (MMR) pathways [5]. Specifically at the Ig locus of activated B cells, BER or MMR processing of AID-lesions does not lead to repair, but can be processed to give rise to DNA mutation and recombination. This complex relationship between the DNA repair processing and AID-lesions is emphasised by the observation that in activated B cells those AID deaminations that occur outside the Ig loci are repaired efficiently [6].

To better characterise the molecular and biochemical mechanisms of AID-induced lesion resolution, we set up an *in vitro* resolution (IVR) assay that allows us to control the substrate DNA, AID, the source of DNA repair proteins, and the various DNA repair pathways themselves. In this system, a supercoiled plasmid is targeted by an AID fusion protein to induce cytosine deaminations. The resulting lesion containing plasmid is added to a cell extract to induce DNA repair. We monitor DNA repair kinetics using biotinylated dNTPs (bio-dNTP), which are incorporated by DNA polymerases. Plasmids that incorporated biotins are isolated using streptavidin magnetic beads and quantitated employing real-time PCR. This is the first description of an assay that incorporates the activity of AID and the subsequent resolution of the lesion by an extract. We describe the specificity of the IVR, highlight the critical steps, rule out biases, and show results that illustrate the advancement and potential of this IVR assay.

## Materials and Methods

### Plasmids

Target plasmid pGL4.31, containing 5 x UAS - GAL4 binding sites, was purchased from Promega. DNA preparation was performed with the Wizard Plus SV Minipreps DNA Purification Systems (Promega) at 4°C [7]. To create the human His-Tagged GAL4-AID expression vector, the DNA-binding domain of GAL4 was inserted into the *Nco*I restriction site of AID-His in pET30 [8]. The catalytic inactive GAL4-AID C87R was created by site-directed mutagenesis.

### Protein Purification

Recombinant GAL4-AID, wild-type and mutant, and AID proteins were purified as previously published [9], with minor modifications. After the overnight expression, the bacterial pellet was resuspended in the extraction buffer (20 mM MES pH 6.0, 300 mM NaCl, 150 mM KOAc, 2.5 mM TECP, 1.6 mM CHAPS, 300 mM L-arginine HCl, 5% Glycerol, filtered and adjusted at pH 6.0) before sonication. The sonicated suspension was centrifuged 165,000 g for 45 min at 4°C, and the supernatant was incubated with Talon Metal affinity resin (Clontech) for 90 min at 4°C. The resin was washed with 5 bed volumes of extraction buffer containing successively 20 mM and 30 mM imidazole, and proteins eluted with 2.5 bed volumes of extraction buffer containing 200 mM imidazole.

### *Xenopus laevis* egg extracts

Preparation of X. *laevis* egg extracts (FE) was performed as described [10].

### *In vitro* resolution system (IVR)

AID-mediated deamination was performed by incubating 1 pmol of GAL4-AID with 0.1 pmol pGL4.31 for 30 min (or indicated time) at 37°C in IVR buffer (40 mM Tris-HCl pH 8.0, 10 mM NaCl, 80 mM KCl, 0.5 mM DTT, 5 µg RNase A). Subsequently, repair of the deaminated plasmid was carried out by the addition of 150 µg of FE supplemented with 5 µg aphidicolin, 0.05 mM dNTP-C (without dCTP and 0.05 mM biotinylated-dCTP (Invitrogen)) or 0.05 mM dNTP-A (without dATP and 0.05 mM biotinylated-dATP (Invitrogen)), and incubated for 30 min (or indicated time) at 23°C. When specified, UNG inhibitor (UGI - New England Biolabs) or PCNA inhibitor T2AA [11] were incubated with the X. laevis egg extracts for 10 min on ice before addition to the reaction. After the repair reaction, free biotin was removed with the Qiaquick PCR purification kit (Qiagen). The DNA was eluted with 100 µl of elution buffer, and 2 µl (further diluted 10 fold) used as ‘input’. The remaining eluted plasmid was subject to Dynabeads M-270 streptavidin magnetic bead (Invitrogen) isolation in TE - 1000 (10 mM Tris pH 8.0, 0.1 mM EDTA, 0.01% Tween-20, 1000 mM NaCl). Mixtures were gently rotated for 15 min at room temperature and left for 5 min on a DynaMag-2 magnet (Invitrogen). The following washes were applied: TE - 500 (10 mM Tris pH 8.0, 0.1 mM EDTA, 0.01% Tween-20, 500 mM NaCl) at RT, TE - 100 (10 mM Tris pH 8.0, 0.1 mM EDTA, 0.01% Tween-20, 100 mM NaCl) at 65°C, TE - 50 (10 mM Tris pH 8.0, 0.1 mM EDTA, 0.01% Tween-20, 50 mM NaCl) at 65°C, and TE - 0 (10 mM Tris pH 8.0, 0.1 mM EDTA, 0.01% Tween-20) at RT. Beads were finally resuspended in 100 µl TE (without Tween-20) and subjected to quantitative real-time PCR. The PCR reaction was performed in 20 µl, containing 2 µl of the bound DNA bead mixture, 10 µl of the LightCycler 480 SYBR Green I master (Roche Applied Sciences) and specific primers (for PCR primers sequence, see Table 1). The reaction was monitored in a LightCycler 480 Real-Time PCR System (Roche Applied Sciences); with the ‘input’ DNA analysed in parallel as reference. C_t_ values for the biotinylated-DNA were correlated to the C_t_ values for the input DNA.

**Table 1:** Sequence of the primers used for the qPCR analyses.

Fragment	Orientation	Name	Sequence
A	For	1830	CATTCTACCCACTCGAAGACGG
A	Rev	1831	AGCCGAACGCTCATCTCG
B	For	1801	CTTCCACCTACCAGGCATCC
B	Rev	1802	CTTACCGGTGTCCAAGTCCAC
C	For	1834	TGCGTATTGGGCGCTCTT
C	Rev	1835	TGGTTCTTTCCGCCTCAGAA
D	For	1836	ATTTCATTTGCGCCATTGCT
D	Rev	1837	CATCAGTTCGTCCAGGGCTT
E	For	1838	AAACTCTAGAACCGGTCATGGC
E	Rev	1839	GCATCGGTCGACAGCATCT
F	For	1840	CCGCTTACCGGATACCTGTC
F	Rev	1841	GAGCGAACGACCTACACCGA
G	For	1871	CGGTGGTTTTTTTGTTTGCA
G	Rev	1872	CGTTCCACTGAGCGTCAGAC
H	For	1844	TCGTCGTTCGGTATGGCTTC
H	Rev	1845	GGCCCTAAGGAGCTGACTGC
I	For	1846	GCACCCAGTTGATCTTCAGCA
I	Rev	1847	CGTGTCGCACTCATTCCCTT
FL	For	1512	GGCCTAACTGGCCGGTAC
FL	Rev	1518	GTCCACCTCGATATGTGC

The IVR results were presented either as relative quantification (fold-change) or absolute (% of input). For fold-change, all samples were compared to their input (defined as the plasmid DNA that was present in the reaction prior to the streptavidin isolation) and then the FE alone sample (or another samples specified) was used as reference and set to one. The fold change was calculated according to the ΔΔ-C_t_ algorithm, represented as: Fold Change  = 2^{−[(Xo−Xi)−(Ro−Ri)]}^, where X - sample; R - reference FE alone; o - output (post bead isolation); i - input (pre-bead isolation). Alternatively, in the % of input analysis the Ct qPCR values of input and output were converted to an absolute amount of DNA based on a standard curve (performed in parallel to the analysis), with the amount of isolated biotinylated-plasmid being expressed as a percentage of the initial amount of plasmid (input).

### Supercoiled and linear DNA gel electrophoresis

Plasmid DNA was untreated or treated either with GAL4-AID, FE, or endonuclease enzyme. After treatment, the topology of the plasmid was assessed using a 0.8% agarose gel. After migration at 5–10 V/cm for 16 h at 4°C the gel was soaked in 1x TBE containing SYBR® Safe DNA gel stain (Invitrogen) for 30 min and plasmids visualised using a Gel Doc system (Bio-Rad).

### Oligonucleotide-based assays

The AID ssDNA oligonucleotide deamination assay was performed as described [12], [13]. Briefly, 2.5 pmol of the 5′-biotin-tagged and 3′-fluorescein-tagged oligonucleotide SPM163 (5′-B-ATTATTATTATTAGCTATTTATTTATTTATTTATTTATTT- FITC-3′) was mixed with RNase A (10 µg) in 10 µl reaction buffer R (50 mM NaCl, 3 mM MgCl2, 40 mM KCl, 40 mM Tris-HCl pH 8.0, 1 mM DTT, 10% glycerol), denatured for 3 min at 90°C and quenched in ice-water. Oligonucleotides were incubated for 30 min at 37°C with 50 ng of recombinant protein. Reactions were stopped and brought to 100 µl total volume in H_2_O. 8 µl of streptavidin magnetic beads (Dynal M270, Invitrogen) were incubated with the oligonucleotides in TE-1000 for 15 min. Beads were collected, washed twice in TE-1000 preheated to 70°C, once in TE at RT, and resuspended in Uracil-DNA Glycosylase (UDG, New England Biolabs) reaction mix, prepared according to manufacturer protocol, and incubated for 1 h at 37°C. Cleavage reactions were stopped by addition of 20 µl 0.4% fushin in formamide and denaturation at 90°C for 3 min. After quenching on ice, samples were resolved on 17.5% TBE-PAGE urea gels at 200 V and visualised using a Typhoon scanner (GE Healthcare) for fluorescence imaging (Filter: 526 SP (532 nm), Laser: Blue 488 nm).

The UNG oligonucleotide assay was performed with oligonucleotides 164 (5′-B–ATTATTATTATTAGUTATTTATTTATTTATTTATTTATTT-FITC-3′) and 166 (5′-AAATAAATAAATAAATAAATAAATAGCTAATAATAATAAT-3′). The oligonucleotides were mixed in 50 mM NaCl, Tris ph 8.0, denatured for 5 min at 90°C, and annealed by slowly cooling to RT (30 min). dsOligonucleotides (containing the mismatch dU:dG) were purified with streptavidin magnetic beads and incubated with the FE for 15 minutes at 23°C. To enhance strand cleavage NaOH (0.3 M) was added. Samples were analysed as above. To inhibit UNG2 present in the extracts UGI was added to the extracts 10 min (on ice) prior to the incubation with the oligonucleotides.

### Statistics

The following statistical analyses have been performed: For those experiments which compare different IVR bar graphs (as shown in the two Figures), standard t-test statistics for each indicated sample groups (n = 3) was performed, and the p values are indicated on the graph above the brackets. For the graph correlating fragment length with IVR activity, the goodness of fit (r^2^), the correlation value (r), and the probability (two-tailed) of the Pearson correlation coefficient were calculated and displayed in the graph (n = 9).

## Results

### Principle of the *In Vitro* Resolution (IVR) assay

**Figure 1:**
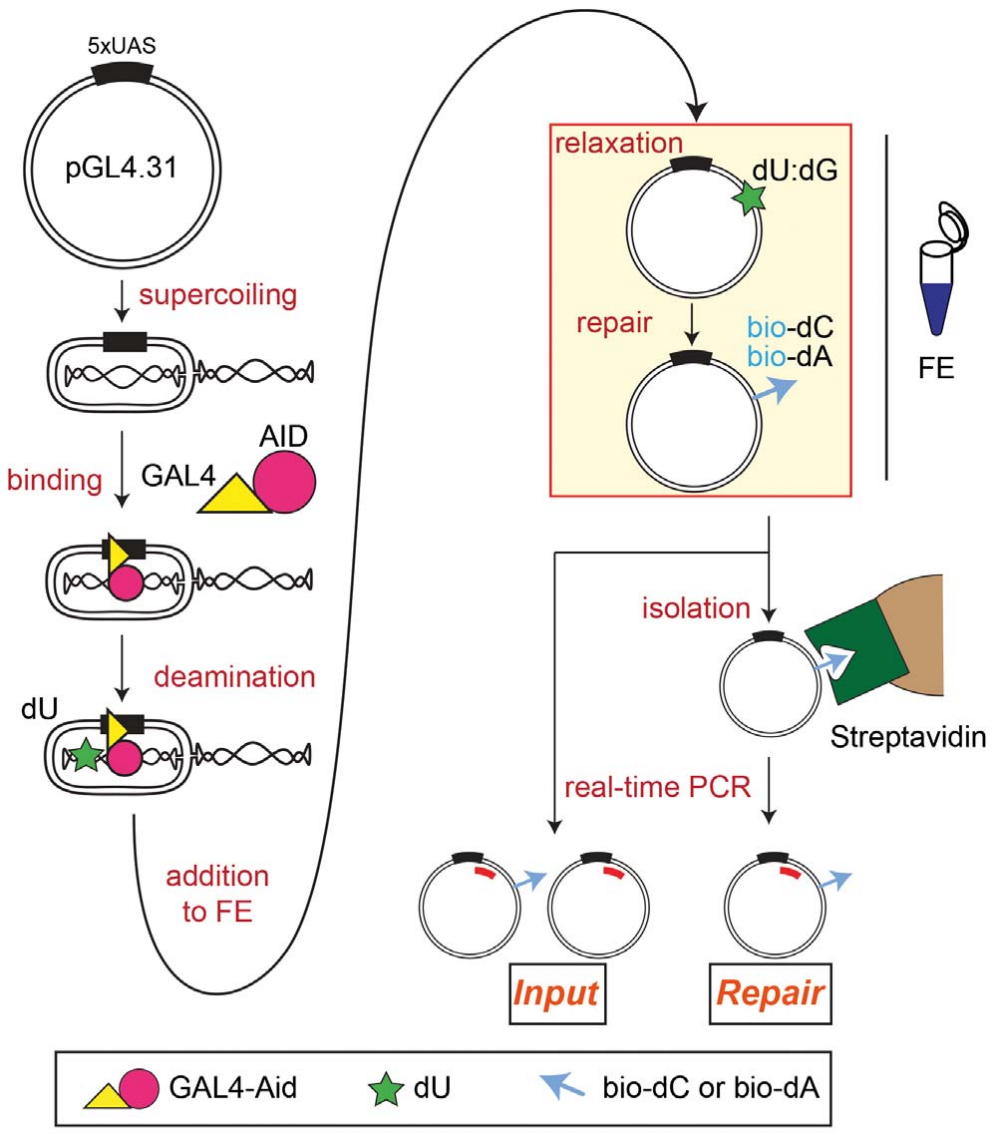
AID-induced lesions repair in vitro by the IVR system. Schematic description of the IVR assay. A supercoiled DNA plasmid (pGL4.31) containing 5 x GAL4 binding sites (UAS) is incubated with a recombinant fusion GAL4-AID protein (G-AID, represented by a yellow triangle and a red circle). Incubation at 37°C deaminates dC to create dU lesions in single stranded DNA (green star). The repair phase (yellow box) - relaxation and lesion repair - is carried out by the addition of frog egg extract (FE) in the presence of biotinylated dCTP (bio-dC) or biotinylated dATP (bio-dA) - (blue arrow), along with normal dNTPs. After DNA repair of the dU lesion the biotinylated-tagged DNA is isolated via magnetic streptavidin beads. Eluted products are subject to quantitative real-time PCR (red bar), and compared to input values of the same reaction prior to streptavidin isolation.

In order to delineate the molecular mechanisms of AID-induced lesion resolution, we designed an in vitro resolution (IVR) system, where AID was targeted to a DNA substrate and a cell extract used for the DNA repair/resolution of the AID-induced lesions. Briefly (**Figure 1**), a bacterially produced GAL4-AID (G-AID) fusion protein is targeted to GAL4 DNA-binding sites (UAS) on a supercoiled (sc) plasmid. The supercoiled topology of the substrate provides both double-stranded DNA (dsDNA) for GAL4 binding and ssDNA required for AID activity; a method which has previously been utilised for the generation of random stretches of ssDNA [14]. Bound AID deaminates dC to dU in the preferred sequence context of WRC. Subsequently, the reaction is added to Xenopus laevis egg extracts (called hereafter frog extract, FE). In the past, FE preparations have been used to study various aspects of cell physiology, including DNA repair pathways such as BER, MMR, or nucleotide excision repair (NER) [15]. DNA topoisomerase I and II within the FE [16] relax the plasmid forming a dU:dG mismatch, which in turn becomes a substrate for various DNA repair pathways. Since all DNA repair pathways acting on dU:dG mismatches require dCTP for lesion processing, supplementing the FE with biotinylated-dCTP (bio-dC) allows for the incorporation of a biotin-tag into the substrate. Importantly, processive polymerase dependent DNA repair pathways not only incorporate dCTP but also other dNTPs; hence addition of biotinylated-dATP (bio-dA) can also serve as a source of biotin incorporation during DNA repair. Biotinylated plasmids are isolated via specific streptavidin bead isolation, with the amount of recovered plasmid being determined by quantitative real-time PCR (qPCR). Correlation of the recovered plasmid to the input plasmid (post-repair and prior to bead isolation) provides a quantitative evaluation of various activities: substrate topology, AID kinetics, FE DNA repair kinetics, and importantly combinations thereof.

### AID-induced lesions initiate resolution

**Figure 2:**
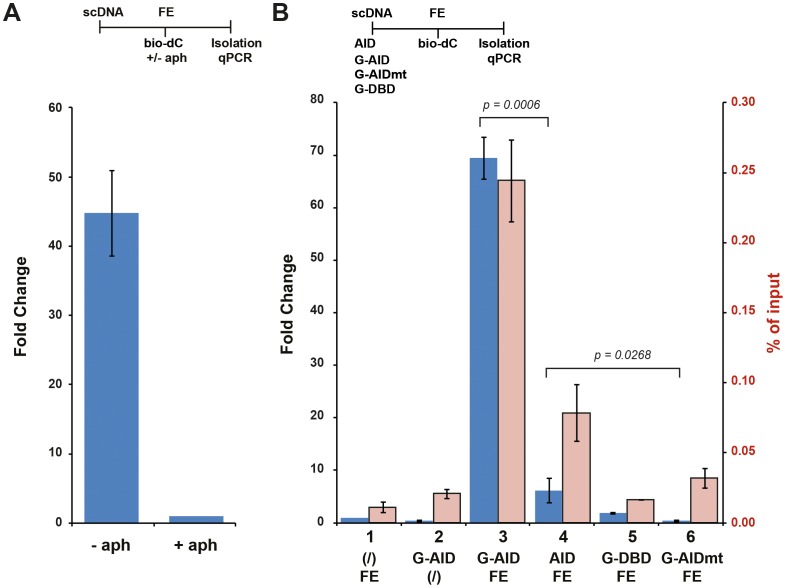
AID-induced damage repair in the absence of replicative polymerases. (**A**) Replicative DNA polymerases were inhibited with aphidicolin (aph) during Xenopus laevis egg extracts (FE) incubation. Supercoiled DNA (scDNA) was added to FE (with bio-dC) in the presence or absence of aph, and biotinylated DNA isolated and quantitated by qPCR. The bars represent fold change as the difference between the qPCR C_t_ value of each sample normalised to the treated (+ aph) sample, which was set to 1. Error bars indicate ± SD (n = 3). Time line of experiment shown above the graph indicates the order of addition of substrates/proteins/nucleotides/extract/etc. or treatments. (**B**) AID-induced lesions are repaired in Xenopus laevis egg extracts. scDNA plasmids were treated (or not - bar 1) with the indicated proteins and then incubated in FE (or not - bar 2), isolated, and quantified by qPCR. The blue bars represent the ratio (fold change) of the amount of recovered plasmids from reactions carried out in the presence of G-AID (bar 3), untagged AID (AID; bar 4), GAL4 DNA binding domain (G-DBD; bar 5), or mutant GAL4-AID C87R (G-AIDmt; bar 6) versus levels of plasmids recovered from reactions that did not contain G-AID (FE alone, set to 1; bar 1). Open pink bars represent the absolute recovery of each treated sample in relation to its input. Error bars indicate ± SD (n = 3). Statistical analysis (t-test) was performed on differences of indicated fold change (brackets), with p values shown. Time line of the experiment is shown above the graph.

Specificity of the IVR system included the avoidance of lesion-independent bio-dC incorporation, possibly due to DNA replication. This was achieved by inhibiting replicative DNA polymerases in the FE using aphidicolin (aph) (**Figure 2A**). In the absence of aphidicolin and any DNA damage the FE readily incorporated bio-dC into the plasmid, while use of aphidicolin reduced the number of plasmids incorporating bio-dC by over 40 fold. All subsequent IVR experiments were performed in presence of aphidicolin.

As shown in **Figure 2B**, performing the reaction with FE alone (bar 1) or G-AID alone (bar 2) did not lead to significant biotinylation of plasmid. On the other hand, when the sc plasmid was incubated with G-AID and then added to FE, an almost 70 fold increase in repair-induced bio-dC incorporation can be observed (bar 3). Using only the GAL4-DNA binding domain (G-DBD - bar 5) indicated that it was not protein binding to plasmid that induced dC incorporation, while use of a catalytic mutant of AID (C87R - G-AIDmt - bar 6) demonstrated the requirement for AID catalysis to initiate DNA repair in the FE. Our results indicate that the IVR is an AID deamination-dependent and DNA repair-dependent assay system.

Representation of the IVR results is shown either as relative (fold change) or absolute (% of input) quantification (**Figure 2B** – blue and pink bars, respectively). Fold change is the activity of each sample as compared to the FE alone (set to 1). % input refers to the absolute amount of plasmid DNA recovered from streptavidin isolation (based on a standard curve) in relation to the input (DNA prior to streptavidin isolation).

Interestingly, when AID without the GAL4 binding domain was used less DNA repair was observed when compared to G-AID (**Figure 2B** - bar 4). In our *in vitro* oligonucleotide deamination assay AID was more active than G-AID on a per molecule analysis (data not shown), hence it is unlikely due to catalytic turnover. Most likely this is due to G-AID being intimately tethered to the substrate thereby reducing the micro-dissociations and hence allow for multiple dUs to be formed on the same substrate [17]. On the other hand we cannot exclude the possibility that G-AID also serves as a binding protein for DNA repair factors in the FE and hence enhanced DNA repair, see our discussion for more detail.

### Plasmid topology

**Figure 3:**
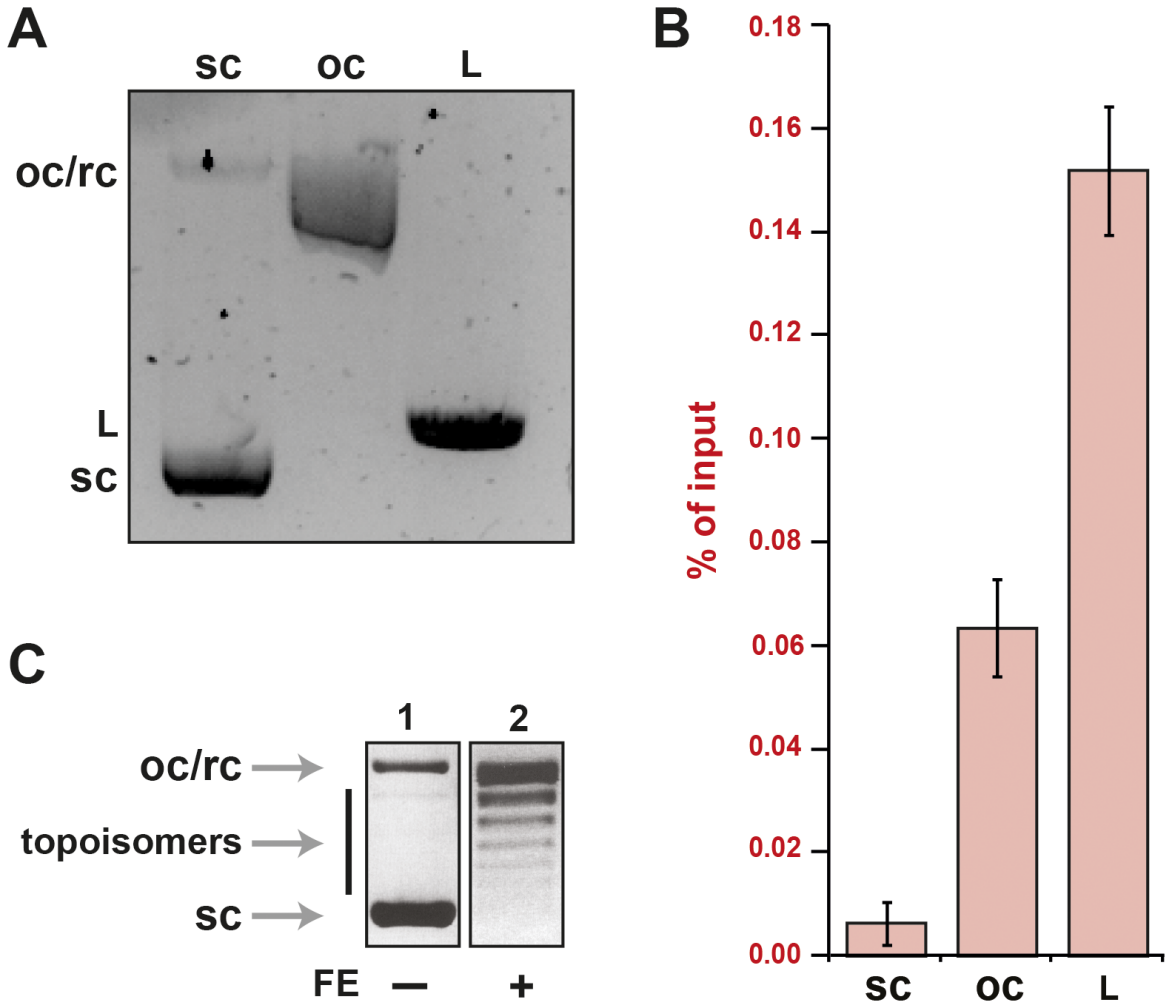
DNA topology during the IVR. (**A**) Various forms of the substrate plasmid were analysed on a 0.8% agarose gel at 5–10 V/cm for 16 h at 4°C. scDNA (sc) migrated faster than linear (L), while nicked open circular or relaxed circular DNA (oc/rc) migrated slowest. (**B**) Each topological form of DNA substrates was subjected to IVR without AID-induced damage. Experiments were done as in Figure 2, and results expressed as % of input. Error bars indicate ± SD (n = 3). (**C**) Modulation of DNA topology due to FE. 1 µg of supercoiled DNA (lane 1) was incubated with FE (lane 2) and analysed as in (A).

In bacteria, most plasmid can be found in the covalently closed circular or supercoiled form, but during plasmid isolation occasional single or double strand breaks result in nicked open circular (oc), relaxed circular (rc), or linear (L) forms of DNA (**Figure 3A**). Since, the latter two forms can lead to DNA repair activation (**Figure 3B**), it is crucial to minimise these structures during purification. Using a modified miniprep purification method, with all steps being carried out at 4°C, resulted in the formation of pure and homogenous supercoiled plasmid [7]. Following AID induced deamination, topological alterations are necessary for the dU to be paired with dG forming a dU:dG mismatch, which is achieved by the topoisomerases within the FE (**Figure 3C**).

### Sensitivity of biotinylated plasmid isolation

**Figure 4:**
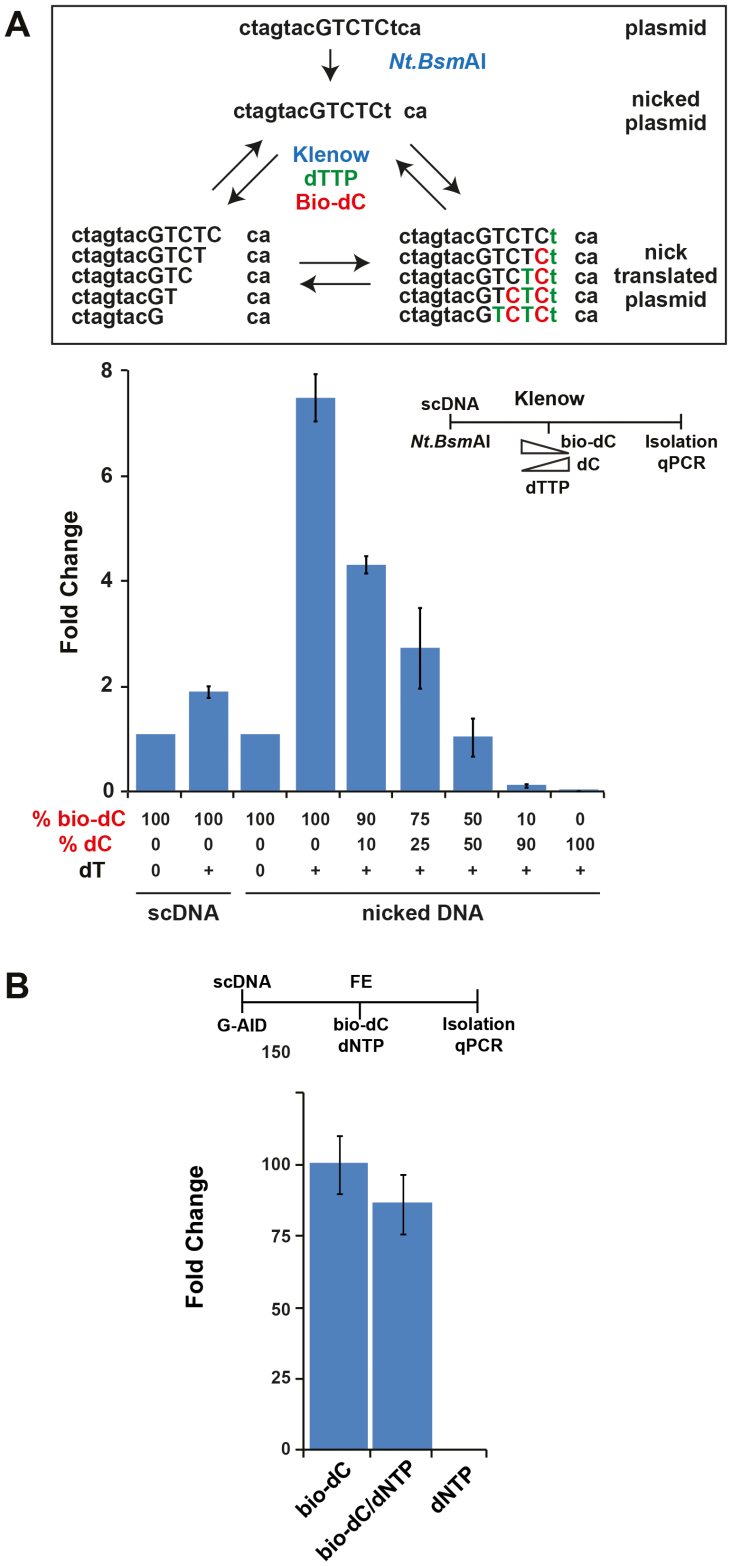
Correlation between lesion size, biotin incorporation, and plasmid recovery. (**A**) Incorporation of 1 or 2 biotinylated dCTP molecules is sufficient to recover the targeted plasmid by streptavidin purification. Schematic of the experiment is depicted at the top of the figure. Plasmids were untreated (scDNA) or nicked at one position with Nt.BsmAI (nicked DNA), followed by treatment with Klenow for 30 min at 25°C in the presence or absence of dTTP (dT) and varying ratios of biotinylated (bio-dC) and normal dCTP (dC). As indicated in the schematic, the lack of dGTP and dATP only allowed for the incorporation of 1 or 2 bio-dC molecules per plasmid. The bars (fold change) represent the difference between the qPCR C_t_ value for each sample before and after the streptavidin purification, normalised to the sample (scDNA or nicked DNA) treated with the Klenow and bio-dC (no dT and set to 1). Error bars indicate ± SD (n = 3). Time line of the experiment is shown above the graph. (**B**) Patch length of incorporated bio-dC does not bias the recovery of plasmids from IVR. The amount of bio-dC incorporation depending on the presence of other dNTPs was monitored by an AID-induced IVR assay. The fold change represents the difference between the qPCR C_t_ value of each sample normalised to the FE-treated sample (no G-AID) that was set to 1.

Isolation of the deaminated and repaired plasmids depends on biotin incorporation. To ensure unbiased recovery, we determined the minimum number of biotin needed for isolation as well as the influence of multiple biotin incorporations per single site. We first assessed whether incorporation of 1 or 2 biotin was sufficient for isolation and amplification. The target plasmid was cut with the nicking endonuclease Nt.BsmAI, which has only one restriction site on the plasmid and recognises the sequence GTCTCN/N (for this plasmid NN  =  TC). Utilising E. coli DNA polymerase I large fragment (Klenow) 3′ → 5′ exonuclease and polymerase activity allowed for the controlled bio-dC incorporation in conjunction with dTTP (**Figure 4A** - schematic). By omitting dGTP and dATP from the Klenow reaction bio-dC incorporation was limited to 1 or 2 per nicked plasmid. Detection of biotinylated plasmid was dependent on the presence of the nick, dTTP to initiate the reaction, and bio-dC (**Figure 4A** - bar graph), demonstrating that a limited number (1 to 2) of biotin incorporations was sufficient for isolation and amplification.

As stated above, AID-induced lesions can be processed via different DNA repair pathways. It was possible that depending on the activated pathway either a single or several nucleotides could be incorporated at the site of the lesion. If multiple biotins per single damage site were more efficiently isolated than a single (or few) biotin, the amplification step would bias one pathway over the other. To this end, a G-AID IVR experiment was carried out with bio-dC in the absence or presence of remaining dNTPs (Figure 4B). From **Figure 4A** it was clear that the presence of dNTPs allows bio-dC incorporation past the initial lesion. The results show that plasmid isolation efficiency, given the same number of damaged sites, was independent on dNTP, and therefore the number of local biotin incorporations did not change recovery efficiency.

### Specificity and activity of GAL4-AID

**Figure 5:**
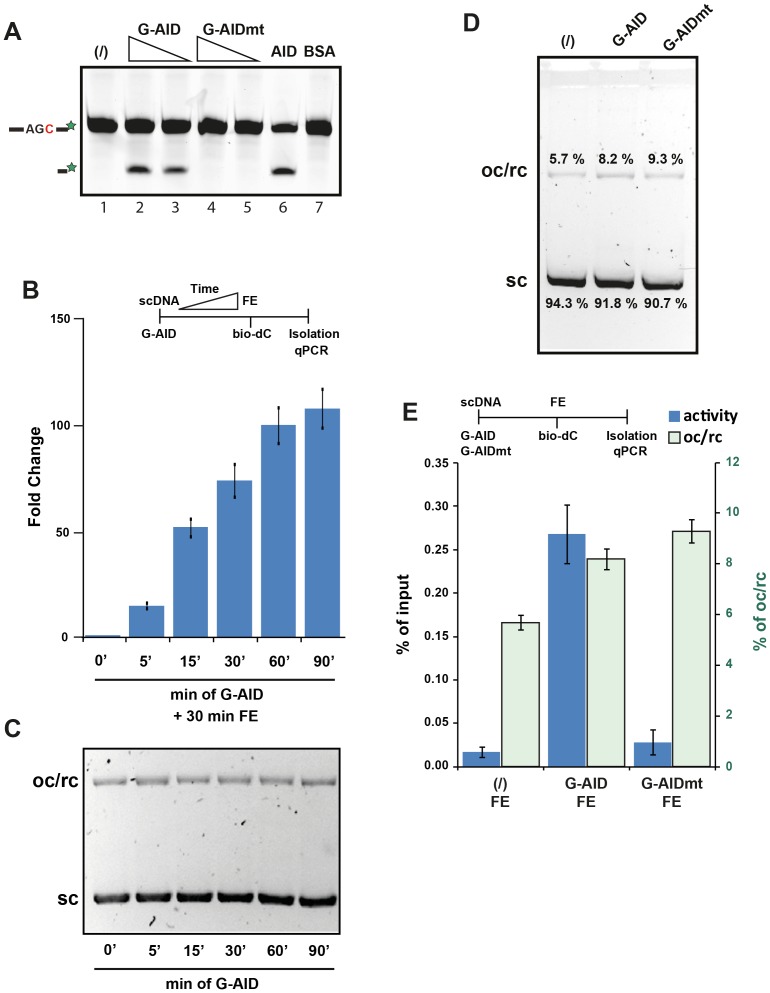
Quality control of AID used for the IVR assay. (**A**) G-AID deaminates cytosine to uracil during in vitro oligonucleotide deamination assay [9]. ssDNA oligonucleotide deamination assay was performed using an oligonucleotide SPM163 containing a single cytosine. Two concentrations (0.05 µg and 0.5 µg) of G-AID and G-AIDmt, as well as untagged AID (0.05 µg) and BSA (1 µg) were incubated with oligonucleotide for 30 min at 37°C, followed treatment with UNG and NaOH, and separated on a 17.5% PAGE gel. (**B**) Time course of G-AID activity during an IVR. G-AID was incubated with substrate for 5 to 90 min (37°C) before addition of the FE (30 min 23°C). Analysis was done as in Figure 2. Error bars indicate ± SD (n = 3). (**C**) G-AID does not induce topological changes on the supercoiled plasmid over time. Samples were processed as in (B), but prior to FE addition they were treated with SDS and proteinase K for 2 h at 56°C and analysed for changes in DNA topology as in Figure 3A. (**D & E**) G-AID and G-AIDmt were incubated with scDNA (as in C) and analysed for topological changes (D) or subjected to an IVR reaction (E). Quantitations of the topological forms of the substrate are shown in green in (E), while IVR results are shown in blue as % of input recovery. IVR analysis was performed as in Figure 2. Error bars indicate ± SD (n = 3).

The aim of the IVR assay is to delineate the DNA repair pathways activated by and acting on AID-induced lesions, therefore it was imperative to validate that the detected lesions were due to AID. GAL4-AID deamination activity was measured by our standard oligonucleotide assay [9]. Here (**Figure 5A**), a labelled oligonucleotide containing a single dC is incubated with G-AID, G-AIDmt, and AID. Subsequently, dUs are detected with DNA glycosylase UNG and abasic site cleavage. GAL4-AID fusion protein efficiently induced dC deamination (lanes 2 & 3) while the catalytic mutant of G-AID did not (lanes 4 & 5).

Performing a time course analysis of G-AID deamination on the target plasmid (5 to 90 min) followed by FE incubation, provided us with initial AID kinetics of the IVR (**Figure 5B**); where after 60–90 minutes saturation was beginning to be reached. The incubation time for all subsequent IVR experiments was set to 30 min to obtain sufficient deaminations while avoiding saturation kinetics. To ensure that the GAL4-AID preparation did not induce other DNA damage due to possible impurities, we monitored plasmid integrity using a gel mobility assay. Samples from the time course where loaded onto an agarose gel (**Figure 5C**), and even after 90 minutes of recombinant protein incubation there were no gross DNA alterations visible.

Catalytic dead mutant of G-AID (G-AIDmt) was purified the same way as wt G-AID, and neither preparation induced gross topological changes (**Figure 5D**). Quantitative analysis of the gels identified a marginal increase in oc/rcDNA formation in the presence of the GAL4 fusion proteins (**Figure 5E** - pink bars) from 5.7% to 8.2% and 9.3%. In the IVR reaction (**Figure 5E** - blue bars), only the G-AID protein and not the G-AIDmt protein induced DNA damage. This indicated that the possible contaminating DNA damaging agents in the recombinant protein preparations did not influence the outcome of the IVR analysis, and only the AID-induced deamination led to biotin incorporation during FE repair activity.

### Specificity and repair activity of the Xenopus egg extract

**Figure 6:**
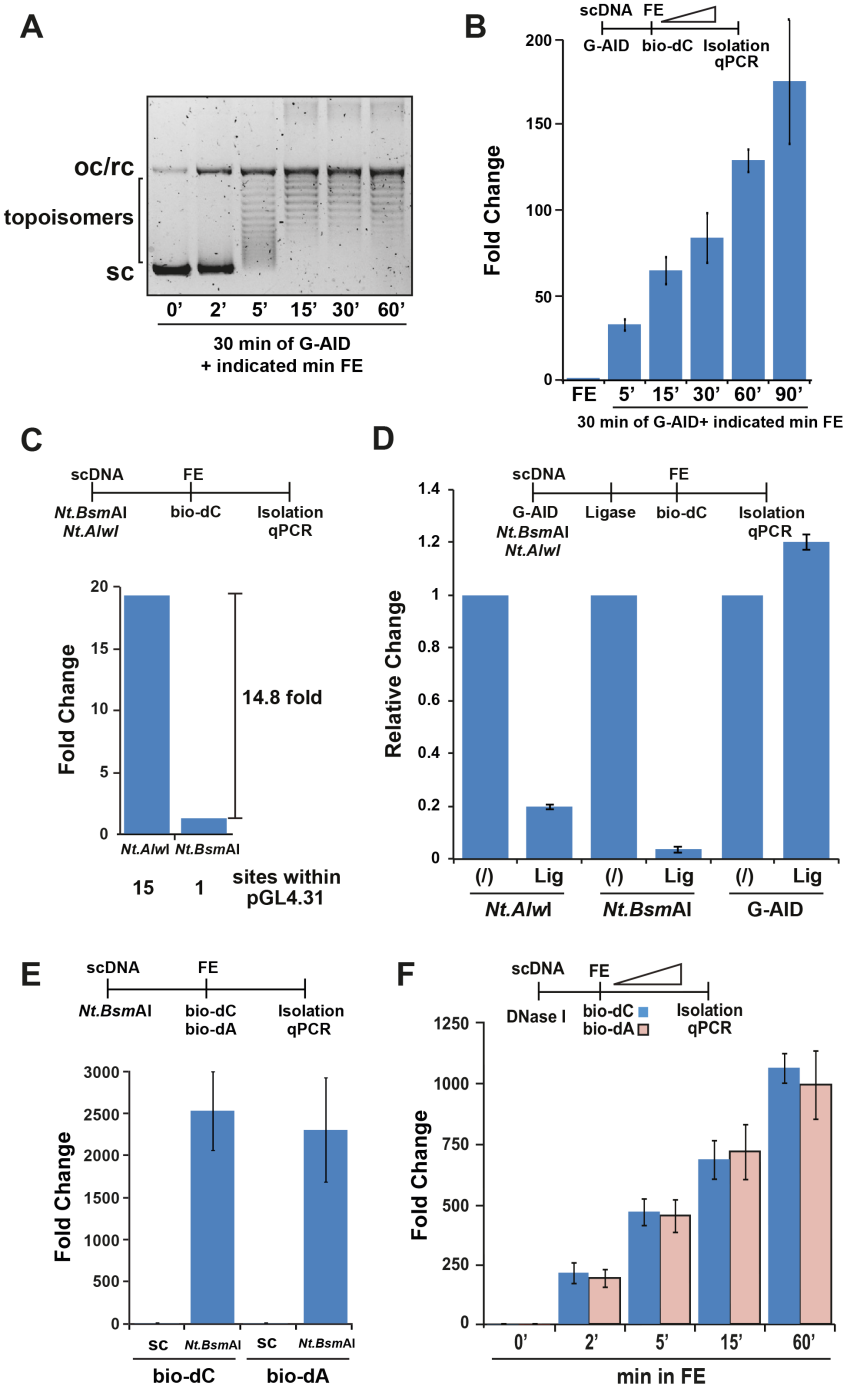
Quality control of FE in the IVR assay. (**A**) DNA topology changes upon FE treatment. scDNA was treated with G-AID, followed by incubation with FE for 0 to 60 min before deproteinisation with SDS and proteinase K at 56°C overnight. Deproteinised DNA was analysed as in Figure 3A. Various topoisomers of the DNA are indicated on the left; oc/rc (open circular/relaxed circular); sc (supercoiled). (**B**) Time course of DNA repair activity during an IVR. G-AID was incubated with scDNA for 30 min 37°C before addition of the FE and incubation for the indicated time (23°C). Analysis was performed as in Figure 2 with untreated sample (no AID) set to 1. Error bars indicate ± SD (n = 3). (**C**) Bio-dC incorporation by FE quantitatively correlates with the number of DNA damages (nicks). FE repair activity was monitored by incorporation of bio-dC after incubation with damaged plasmid (nicked with Nt.AlwI or Nt.BsmAI, which cut the plasmid 15 or 1 times, respectively). Analysis was performed as in Figure 2 with untreated sample (no FE - not shown) set to 1. (**D**) Repair specificity in FE. Repair activity was monitored by the incorporation of bio-dC after incubation of the nicked (Nt.AlwI or Nt.BsmAI) or re-ligated nicked (Lig - T4 ligase) plasmid with FE. The effect of T4 ligase on a G-AID-treated scDNA plasmid sample was also tested. Analysis was performed as in Figure 2 with untreated sample (no ligation) set to 1. Error bars indicate ± SD (n = 3). (**E**) The incorporation of bio-dC or bio-dA from a nick by FE is equivalent. FE DNA repair activity was monitored by incorporation of bio-dC or bio-dA after incubation of either a supercoiled (scDNA) or nicked (Nt.BsmAI) plasmid. Analysis was performed as in Figure 2 with untreated sample (no FE - not shown) set to 1. Error bars indicate ± SD (n = 3). (**F**) Time course of FE activity on a nicked damaged plasmid. DNase I (0.001 U) treated scDNA was incubated with FE for 0 to 60 min (23°C) in the presence of bio-dC or bio-dA. Analysis was performed as in Figure 2 with untreated sample (no FE - not shown) set to 1. Error bars indicate ± SD (n = 3).

In the FE, the initial step during repair resolution of AID-induced damage is plasmid relaxation, which leads to double-stranded formation and thereby a dU:dG mismatch. As shown in the **Figure 6A**, G-AID treated plasmid showed initial relaxation within 2 min, plateauing by 15 min. Although, the small amount of oc/rc plasmid that is generated by the FE after 2 min could have activated bio-dC incorporation, we disregarded it for the following reasons: 1) Even 100% oc/rc conversion would lead to less IVR activation then G-AID alone (compare % input recovered in Figure 2B to Figure 3B); 2) The oc/rc could be repaired in the FE and hence become undetectable; 3) IVR assays involving AID activity are measured against FE alone (or AID mt plus FE) and hence background activity would be controlled for. The recovery of biotinylated plasmid (i.e. repaired) from the same IVR reaction was detected after 5 min, but plateaued long after the relaxation plateau (>90 min, **Figure 6B**), indicating that relaxation preceded DNA repair. To avoid saturation kinetics during the IVR the FE reaction was limited to 30 min.

To establish if the FE repair activity correlates with the number of damage sites, we nicked the substrate plasmid with nicking-endonucleases Nt.AlwI or Nt.BsmAI, which nick the plasmid 15 and 1 times, respectively. The recovery of plasmid from FE resolution demonstrated a linear correlation with the amount of damage (**Figure 6C**), with Nt.AlwI-nicked plasmids recovered 15 times more efficiently than Nt.BsmAI-nicked plasmids (**Figure 6C**). Because nicked plasmids are easily sealed with DNA ligase, addition of T4 ligase after the nicking reaction reduced the IVR results 80–95% (**Figure 6D**). In contrast, plasmid treated with G-AID was not sensitive to T4 ligase treatment, which further substantiates that the IVR's DNA repair activity is due to cytosine deamination after AID treatment (**Figure 6D**).

If DNA damage is repaired via different DNA repair pathways, then different biotinylated dNTPs could be incorporated to varying extent. We monitored the ability of the FE to incorporate either bio-dC or bio-dA from a nicked plasmid. We either introduced a single nick with the nicking endonuclease Nt.BsmAI or multiple nicks by treatment with a low concentration of DNase I (0.001 unit), followed by IVR in FE. Incubation of the single nicked plasmid showed a similar recovery with both types of biotinylated dNTP (**Figure 6E**). Analogously, DNase I-treated plasmid showed identical time-kinetic profiles with bio-dC and bio-dA (**Figure 6F**). Therefore, IVR assays can be performed with various biotinylated dNTPs.

### Plasmid substrate properties for the IVR

**Figure 7:**
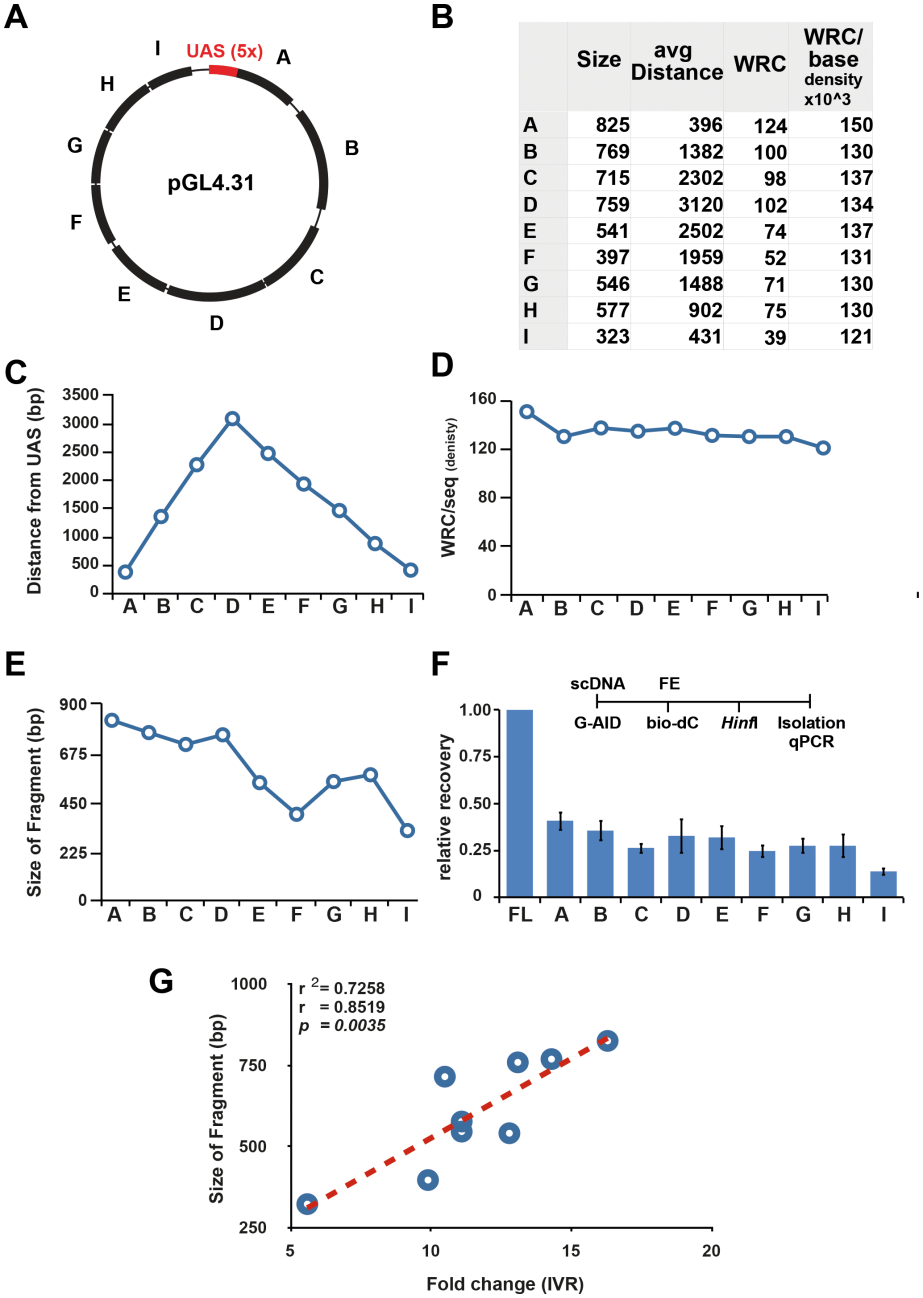
Substrate characterisation for IVR activity. (**A**) Schematic of plasmid used for IVR. GAL4 binding sites (5 x UAS) in red and fragments (A–I) which are monitored by qPCR are indicated. All fragments are flanked by HinfI sites. (**B**) Table indicating size, distance from UAS, and average number of WRC per sequence for each fragment. (**C**–**E**) Graphic representation of data from the table in (B). (**F**) AID-induced IVR fragment analysis. The substrate plasmid was cleaved with HinfI after FE repair prior to streptavidin isolation (see timeline). Analysis was performed as in Figure 2 with uncut (full length - FL) plasmid sample set to 1. Error bars indicate ± SD (n = 3). (**G**) Correlation of fragment length and IVR activity. The fold change values from (F) were plotted against the fragment length and a line of best fit generated. The r^2^ value indicated a positive correlation of length and IVR recovery.

In the past, biochemical analysis of DNA deaminases was limited to using oligonucleotides. This highly artificial substrate presented the enzyme with its preferred substrate-domain, cytosine, but all possible contextual information (sequence and chromatin) was lost. The use of a supercoiled plasmid is the first step to provide a more physiological target for DNA deamination [14]. A supercoiled plasmid can provide information on linear distance, single-strandedness, sequence context, or substructures of DNA. To fully utilise this advantage the plasmid was analysed in fragments, where the target plasmid was subject to restriction digestion (HinfI) after G-AID incubation and IVR, but prior to streptavidin isolation. Eluted DNA was subject to 9 different qPCR reactions, encompassing fragments A–I (**Figure 7A & F**). The distance from the GAL4-AID binding site (UAS) did not influence the efficiency of AID targeting events (**Figure 7B, C & F**), as fragment D (the furthest from the UAS) was as efficiently targeted by G-AID as fragment H. Since all fragments contained approximately the same density (number of WRC/bases) of AID targets (**Figure 7B & D**), longer fragments containing more WRC motifs had a higher chance of being targeted by AID for deaminations. As seen in **Figure 7G**, plotting fragment size (**Figure 7E**) vs. the IVR activity (fold change - derived from **Figure 7F**) demonstrates a statistically significant correlation between fragment length (total WRC number) and IVR activity. This confirmed our previous analysis using the nicking enzymes (**Figure 6C**), where we detected a good correlation between number of damaged sites on the plasmid and efficiency of recovery from IVR. These results also indicate that the GAL4-AID fusion protein activity is directly correlated to the number of WRC sites, and hence reproduces AID's targeting preference for WRC.

### AID-induced lesions are resolved via several DNA repair pathways

**Figure 8:**
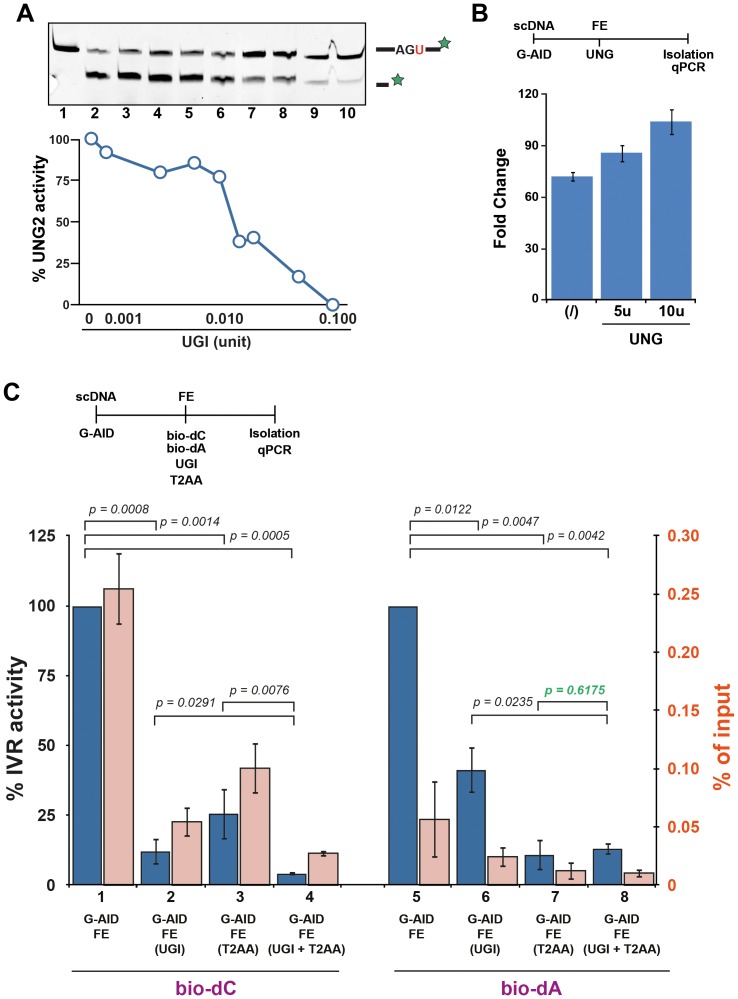
Multiple DNA repair pathways resolve AID-induced lesions. (**A**) Inhibiting the UNG activity of FE with UGI. Top - Increasing amounts of UGI were added to the FE during oligonucleotide based deglycosylation [13]. After NaOH treatment the fragments were separated using PAGE. Bottom - Quantification of deglycosylation activity of UNG2. Activities of lane 2 and 10 were set to 100% (no inhibition) and 0% (maximum inhibition), respectively. (**B**) UNG2 activity in FE is near saturation. 5 or 10 units of recombinant UNG2 were added to the FE reaction during the IVR reaction. Analysis was performed as in Figure 2 with untreated (no G-AID - not shown) set to 1. Error bars indicate ± SD (n = 3). (**C**) DNA repair pathway inhibitors alter IVR of AID lesions. AID-induced damaged plasmids were subject to FE IVR in the presence of bio-dC (bars 1–4) or bio-dA (bars 5–8). BER inhibitor UGI was added to the FE either alone (bars 2 & 6) or in combination with PCNA inhibitor T2AA (bars 4 & 8), while T2AA was also added alone (bars 3 & 7). Analysis was performed as in Figure 2 with untreated FE (bars 1 & 5) set to 100% (blue bar). For absolute comparisons, analysis was also shown as % of input (pink bars). Error bars indicate ± SD (n = 3). Statistical analysis (t-test) was performed on differences of indicated fold change (brackets), with p values shown.

During SHM of the Ig locus, AID-induced lesions can be resolved through the recruitment of DNA repair factors from BER, MMR, and trans-lesion synthesis (TLS). Although some of the key proteins of SHM are known, it is not clear how they interact to resolve the lesions, especially how the initial uracil can lead to mutations away from the target site. With the IVR, the first biochemical approach to study a complex system such as SHM and DNA repair, one can begin to dissect the details and reveal how DNA repair networks interact on various lesions. Because the AID-induced lesion is most likely to be recognised by BER, we characterised FE for UNG activity. Using a previously described oligonucleotide deglycosylation assay [13], the FE UNG activity was determined to be 1.74 fmol/min/µg. Most eukaryotes express a number of different deglycosylases which can recognise uracil in DNA (UNG2, SMUG, TDG, or MBD4 - [18]) with UNG2 being the predominant enzyme present during DNA replication. UGI is a small phage peptide known to inhibit all known UNG2 enzymes [19]. Titrating UGI in the FE (**Figure 8A**) showed that 0.1 units of UGI were able to inhibit all of the UNG2 within FE, which constituted 88.7% of the UNG activity leaving 11.3% of the UNG activity for SMUG, TDG, and MBD4. To ensure that the UNG activity in the FE was not limiting during the IVR resolution phase, we added recombinant UNG2 to the IVR post AID-induced lesion generation (**Figure 8B**). Although, we could detect a slight increase with 5 and 10 units of UNG2 (more than 10 fold of the activity in the FE itself), we decided not to artificially add UNG2 during the resolution phase of the IVR in subsequent experiments.

We reasoned by inhibiting the key protein of BER, UNG, the IVR would reveal other DNA repair activities acting on the dU:dG lesion. Furthermore, analysis of short-patch BER (SP-BER - only acting on the lesion itself) can be separated from long-patch (LP-BER - resynthesising 4–10 bases away from the lesion), and indeed by those from any DNA repair requiring processive polymerase activity, by the use of bio-dC and bio-dA. Bio-dA incorporation only occurs in DNA repair pathways utilising a processive DNA polymerase, while bio-dC can be utilised by any repair system.

IVR recovered plasmids in presence of either bio-dC or bio-dA (**Figure 8C** - bar 1 & 5), indicating that at least two different DNA repair pathways could act on the lesion, one with a processive and one with a non-processive activity. Although the absolute levels of dA and dC incorporation are different (pink bars), for DNA repair pathway analysis we used relative comparisons (blue bars). Inactivating BER with UGI decreased bio-dC recovery by 4 to 5 fold, (bar 2), while the recovery of bio-dA labelled plasmids was inhibited by nearly 2 fold (bar 6). These data indicated that SP-BER, LP-BER, and a processive DNA repair pathway were recognising AID-induced dUs in the FE and processing them for repair. Alternatively, the difference in bio-dA and bio-dC incorporation could also be derived form residual BER activity not dependent on UNG2, such as TDG, SMUG, or MBD4 [20], [21].

To control for processive polymerase dependent repair we took advantage of another inhibitor, T2AA [11]. This small molecule inhibitor acts directly on PCNA, a trimeric sliding clamp that enhances processivity of DNA polymerases and plays a key role in several DNA repair pathways [22]. T2AA impedes binding of all the proteins that interact with PCNA through the PCNA-interacting-protein motif [23], and hence can inhibit MMR as well as LP-BER. When T2AA was added to the FE (bar 3), plasmid recovery of bio-dC was significantly reduced, with the remaining activity attributable to SP-BER. A combination of UGI and T2AA would inhibit BER and MMR-like activity leaving almost no FE IVR activity to act upon AID-induced lesions (bar 4). These results were confirmed using bio-dA in IVR, as only processive DNA polymerase repair activity can be detected (bar 7). As expected, a combination of UGI and T2AA did not further reduce the IVR activity in the FE (bar 8), confirming that AID-induced lesions resolution is in part dependent on PCNA and therefore involves processive polymerase activity.

Altogether, the inhibition results show that AID-induced lesions can be resolved by different DNA repair pathways, including UNG-dependent SP-BER or LP-BER as well as MMR-like. Furthermore, our IVR assay presents a biochemical alternative to genetic analysis of DNA deaminase induced lesion resolution.

## Discussion

The outcome of immunoglobulin diversification is dependent on the synergy between AID deamination and DNA repair pathways and their proteins. Because some of the key DNA repair proteins involved in Ig diversification are also proteins critical for general genome stability, it is reasonable to hypothesise that modifications of the canonical DNA repair pathways occur during the processing of the AID-induced dU lesions. This dichotomy between proper repair and inducing genomic alterations is also the limiting factor for genetic analysis of Ig diversification and requires biochemical approaches to understand the downstream events of AID-induced lesion resolution.

To date there are a few in vitro systems capable of dissecting individual aspects of the Ig diversification process, but none that encompass lesion generation by AID and lesion resolution by defined extracts. Previous in vitro assays characterising the processing of AID-induced lesions used a plasmid containing synthetically introduced mutations [24], [25], thereby removing AID from the actual process. Other systems used plasmid based AID deamination assays, but the readout was dependent on *E. coli* genetics [14], [26], [27], [28]. The IVR system is the first to combine both biochemical aspects in one assay, providing the possibility to dissect key events at the chromatin level, including substrate accessibility, AID targeting, AID kinetics, DNA repair pathway choice, and repair kinetics. While the IVR's reliance on GAL4 binding prohibits interpretation of AID's DNA binding kinetics (e.g. micro-dissociations and associations [17]), a recently published in vitro assay using cytoplasmic and nuclear extracts from various cell types (including B cells) [29] complements our IVR. Pham et al. relied on synthetically placing dU lesions in a plasmid, treating the DNA damage in various extracts in the absence of BER, and then using *E. coli* genetics to look for lack of DNA repair (i.e. mutations due to AID). Since the IVR is identifying DNA repair rather than mutation, a combination of the two assays will become a valuable tool. Furthermore, the IVR recapitulates in vitro known in vivo observations, and provides an efficient approach for studying Ig diversification. Our findings can be summarised as follows: 1) the supercoiled plasmid is a target for AID beyond being a substrate for the IVR (**Figure 2A**); 2) the deamination efficiency of AID is not altered by the fusion with GAL4 (**Figure 5**); 3) catalysis by AID is a requirement for the initiation of the IVR to proceed (**Figure 2B**); 4) the substrate's topology can alter the IVR resolution (**Figure 7**); and 5) multiple repair pathways are activated upon AID-induced lesions recognition (**Figure 8**).

As mentioned, there are a number of physiological aspects of DNA damage and repair that the IVR can address. The use of UGI inhibitor demonstrated the involvement of UNG-dependent BER in the resolution of AID-induced lesion, either SP-BER or LP-BER. Furthermore, the incorporation of bio-dA and the requirement of PCNA (treatment with T2AA inhibitor) would only occur during processive polymerase dependent repair, either LP-BER or MMR-like. Importantly, the inhibition by T2AA also reflects the known involvement of PCNA during SHM in vivo [30], [31]. The partial inhibition of bio-dA incorporation by UGI indicates that UNG-dependent LP-BER is involved. The identification and separation of function into SP-BER, LP-BER, and a MMR-like pathway are a direct reflection of how AID-induced lesions can be processed in vivo during Ig diversification, with the UNG-dependent BER [32], [33] and MSH2/6 MMR [34], [35], [36], [37], [38], [39], [40] pathways playing critical roles during SHM.

The IVR thus provides a fertile ground for the exploration of all aspects of DNA damage induced lesion resolution. Future analyses include: use of other DNA damaging methods (chemical modification, radiation-induced modification, DNA modifying enzymes, restriction enzymes, other DNA deaminases - APOBECs), use of modified substrates (defined structured sequences, general and specific base modifications, nucleosome and chromatin containing), kinetics of DNA damaging protein (i.e. AID - via modifications, mutations, or cofactor associations), extract composition (extract from human cells of different sources, cell cycle specific extracts, depleted extracts, activated extracts, inhibited extracts, pharmacologically altered extracts), and modified DNA as a damaged substrate (denatured, linearised, deproteinated, base modified, nucleosome chromatin formed). Xenopus egg extracts support chromatin assembly in vitro [41] thereby providing an environment in which the effect of AID-induced lesion resolution can be monitored in the context of a chromatin template. Because of its in vitro nature, the IVR is a novel platform for drug-screening programs interested in finding new inhibitors for AID/APOBECs and the DNA repair pathways associated with the lesion resolution, or the development and validation of novel DNA repair pathway inhibitors.

While AID is required to initiate Ig diversification, unregulated off-target activity can result in DNA mutations and translocations of oncogenes and tumour suppressors [42]. How the balance between beneficial and pathological mutations is controlled is not known and subject to intense research in many laboratories. Past work by us and others implied AID to be retained with the RNA pol II transcription complex, presumably after promoter escape [43], [44], which would provide substrate access of single stranded DNA as well as a possible targeting mechanism. Because of the AID-associated factors identified within this complex, it is currently thought that during Ig diversification RNA pol II transcription is paused or stalled within the Ig gene, allowing for increased presence of AID at its substrate, thereby leading to mutations of the dU rather than repair [44]. This hypothesis seems to be substantiated using the IVR, since we can demonstrate that if AID is retained at its own lesion then AID can induce alterations of key DNA repair proteins, and change the efficacy of DNA repair (K. Willmann, et al. personal communication and **Figure 2B** bar 3 vs. bar 4). This novel function of AID, altering DNA repair, was possible to detect using the IVR, as we avoided the complication of AID targeting and did not separate AID-deamination from DNA repair lesion resolution.

Thus, as a novel tool, the IVR can be used to distinguish among DNA repair pathways using a biochemical approach complementing *in vivo* and genetic analysis. Apart from analysing AID activity, the IVR is well suited for characterising other DNA deaminase pathways, or any DNA associated protein initiating or altering DNA repair functions. Thereby the IVR will cross research fields such as immune diversification, viral inactivation, and epigenetics, with chromatin structure and DNA repair.
